# Capability of leaf interdigitation with different inverse planning strategies in Monaco: an investigation of representative tumour sites

**DOI:** 10.1186/s13014-016-0655-1

**Published:** 2016-06-17

**Authors:** Jinghao Duan, Xiangjuan Meng, Tonghai Liu, Yong Yin

**Affiliations:** Department of Radiation Oncology, Shandong Cancer Hospital and Institute, 440 Jiyan Road, Jinan, 250117 China; Shandong Eye Hospital, Shandong Eye Institute, Shandong Academy of Medical Sciences, 372 Jingsi Road, Jinan, 250021 China

**Keywords:** Leaf interdigitation, Inverse treatment technologies, MLC

## Abstract

**Purpose:**

The aim of this study was to experimentally assess the dosimetric impact of leaf interdigitation using different inverse treatment strategies for representative tumour sites and to identify the situations in which leaf interdigitation can benefit these tumour sites.

**Material and methods:**

Sixty previously treated patients (15 nasopharyngeal carcinoma (NPC), 15 multiple brain metastasis (MBM), 15 cervical cancer and 15 prostate cancer) were re-planned for volumetric modulated arc therapy (VMAT), sliding window IMRT (dMLC) and step-and-shoot IMRT (ssIMRT) with and without leaf interdigitation. Various dosimetric variables, such as PTV coverage, OARs sparing, delivery efficiency and planning time, were evaluated for each plan. In addition, a protocol developed by our group was applied to identify the situations in which leaf interdigitation can achieve benefits in clinical practice.

**Results:**

Leaf interdigitation produced few benefits in PTV homogeneity for the MBM VMAT plans and NPC ssIMRT plans. For OARs, sparing was equivalent with and without leaf interdigitation. Leaf interdigitation showed an increase in MUs for dMLC plans and a decrease in MUs for ssIMRT plans. Leaf interdigitation resulted in an increase in segments for dMLC plans and a decrease in segments for NPC and MBM ssIMRT plans. For beam on time, leaf interdigitation showed an increase in MBM dMLC, NPC ssIMRT and prostate ssIMRT plans. In addition, leaf interdigitation saved planning time for VMAT and dMLC plans but increased planning time for ssIMRT plans.

**Conclusion:**

Leaf interdigitation does not improve plan quality when performing inverse treatment strategies, regardless of whether the target is simple or complex. However, it influences the delivery efficiency and planning time. Based on these observations, our study suggests that leaf interdigitation should be utilized when performing MBM VMAT plans and NPC ssIMRT plans.

**Electronic supplementary material:**

The online version of this article (doi:10.1186/s13014-016-0655-1) contains supplementary material, which is available to authorized users.

## Background

Multileaf collimators (MLCs) are modulation devices that are a key invention in the history of radiation therapy [1]. MLCs enable irregular segments and serve as a shielding device that improves target dose conformity. The properties of MLCs, such as leaf width, movement velocity and tongue-and-groove design, influence the dosimetric quality and effectiveness in various IMRT strategies for different types of cancer. Several researchers previously published studies on the dosimetric effects of these features of MLCs. Leaf width is available in increasingly thinner widths on the open market. Several planning studies were performed to evaluate the dosimetric impact of MLC leaf width [2–9]. Treatment plans using thinner leaf width MLCs can result in greater sparing of organs at risk (OARs). These plans have been delivered with more segments and monitor units. However, the clinical benefit is not unequivocal [6, 7]. Moreover, other phenomena were found in the published literature because the thinner leaf provided improved target coverage and dose gradients for small volumes [8, 9]. In addition, the smaller MLC leaf width was more effective for complex-shaped targets [8]. Different leaf velocities have larger impacts on dMLC plans. Hilke Vorwerk et al. [10] found that high leaf velocity showed the best protection for OARs although higher than 3.0 cm/sec leaf velocity is not mechanically applicable. They recommended an optimal leaf velocity of 2.5 cm/sec. The tongue-and-groove design is a feature of MLCs that reduces leakage between leaves. A published study demonstrated that the tongue-and-groove effect was clinically insignificant for multiple-field IMRT because of the smearing effects of individual fields [11]. To minimize the contribution of the tongue and groove effect in dual-arc VMAT plans, several scholars proposed two coplanar arcs with the collimator rotated to some degree [12–14].

**Fig. 1:**
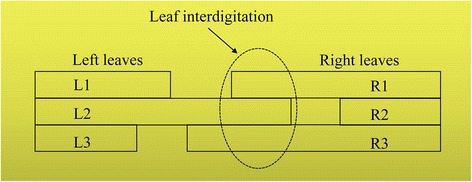
Schematic diagram of leaf interdigitation. The left and right leaf pairs of a row can overlap with the right and left pairs of the adjacent row, respectively

Figure 1 shows that as a property of MLCs, leaf interdigitation refers to the end of a trailing leaf extending past the end of an adjacent leading leaf. Namely, opposing leaves of adjacent rows can overlap [15, 16]. With interdigitation, MLC capabilities have caught up with the ability of the treatment planning system to create island fields in difficult cases. Theoretically, the complex geometry segments increase the degrees of freedom for generating high quality plans. From this perspective, interdigitation allows easy planning of IMRT techniques. However, leaf interdigitation has a limitation, in that it may cause a leaf collision and increase the wear of MLCs. In fact, several researchers with different views have published regarding this topic [4, 17–19]. However, currently, experimental investigations of the ability of MLC leaf interdigitation with inverse planning techniques for different types of tumour sites are scarce, although interdigitation is widely applied in 3D-CRT techniques.

In this study, we further evaluated this topic and assessed the dosimetric advantages and effectiveness of leaf interdigitation with volumetric modulated arc therapy (VMAT), sliding window IMRT (dMLC) and step-and-shoot IMRT (ssIMRT) for four types of representative tumour sites. We expect to determine suitable conditions in which leaf interdigitation is superior to leaf non-interdigitation. Our research may provide useful guidelines for selecting reasonable treatment methods in clinical practice.

## Material and methods

### Patients

To experimentally evaluate the effect of MLC leaf interdigitation, computed tomography data sets from the four following tumour sites identified at our institution between 2014 and 2015 were randomly selected for this study: nasopharyngeal carcinoma with simultaneous integrated boosts (NPC-SIB) (15 patients), multiple brain metastasis (MBM) (15 patients, 3 to 6 isolate targets), cervical cancer (15 patients) and prostate cancer (15 patients). The four tumour sites represent four characteristic PTV shapes, rather complex target volume, multi-isolated target volume, horseshoe-shaped target volume and spherical (simple) target volume. The ethics committee of Shandong Cancer Hospital and Institute approved this study. In this study, the same dose prescription was used for each case to better investigate the effect of leaf interdigitation in different radiotherapy approaches. The PTV definition and dose constraints for the targets and OARs are summarized in Table 1.

**Table 1:** Target description, PTV prescription and OARs for tumor sites and OARs

	NPC-SIB	MBM	Cervical	Prostate
Target description	PTV_70_ = GTV + 5mm; PTV_60_ = HR + 5mm; PTV_54_ = LR + 5mm	PTV = CTV + 7mm; 3 to 6 isolate targets	PTV = ITV + 10mm	PTV = CTV + 7mm
Prescription	PTV_70_:70Gy to GTV and lymphadenopathy; PTV_60_:60Gy to HR; PTV_54_:54Gy to LR; 35 fractions	60Gy to PTV; 30 fractions	50Gy to PTV; 25 fractions	76Gy to PTV; 38 fractions
OARs	Eyes; brainstem; lens; spinal cord; parotids; optic nerves; etc	Eyes; brainstem; lens; parotids; optic nerves; etc	Bladder; rectum; femurs head; small bowel; etc	Bladder; rectum; femurs head; small bowel; etc

Abbreviations: *HR* high risk lymphatic regions, *LR* low risk regions

### Planning techniques and beam setup

A total of 360 plans were generated for the planning study, in which 6 plans were generated per patient using dual arc VMAT, dMLC and ssIMRT with or without leaf interdigitation. Elekta synergy linear accelerator was used in this study. The linac was equipped with the MLCi2 MLC, and the dose rate was 600 MU/min. The MLCi2 with the interdigitation feature has 40 pairs of leaves with a 10 mm width at the isocentre. All plans were generated by an expert planner using our clinical TPS (Monaco version 3.3, Elekta AB, Sweden). The experiments were conducted on an HP Z820 workstation with an Intel Xeon E5-2670 processor at 2.6 GHz with 32 GB RAM and 32 cores.

The expert planner manually designed 3 plans for each patient with the clinical TPS carrying leaf interdigitation permission as follows: dual arc VMAT, dMLC and ssIMRT with 7–9 equiangular beams. The OAR doses were restricted according to RTOG0225, ROG0615 and QUANTEC. In this study, we adopted Sharfo’s methods to avoid the consecutive generation of plans for a single patient [20]. We altered the objective function of DVH and priorities during optimization to achieve the best results for each leaf interdigitation plan. If plan quality levelled off and no significant further improvement was expected, we stopped altering the objective function of DVH and priorities on the plans, and there was no time limit. For acquiring the planning time, the leaf interdigitation plans were generated again using the last objective function of DVH and priorities without previous fluence or segmentation. When the first step was complete, leaf interdigitation permission was abandoned by modifying the TPS data. Then, for a fair comparison, leaf non-interdigitation plans (without) were generated on the condition that the planning parameters of the machine, such as the priorities and dosimetric objectives, were identical to those of the leaf interdigitation plans (with). The leaf non-interdigitation plans were generated without any interference of the fluence and segmentation of the leaf interdigitation plans. After calculation, to eliminate the dependence of the plans on tumour coverage, each plan was normalized such that the prescription dose covered at least 95 % of the PTV.

### Plan evaluation criteria

PTV and main OARs metrics extracted from dose-volume histograms (DVHs) were evaluated. The PTV, maximum dose (dose received by 2 % of the target volume, D_2%_), minimum dose (dose received by 98 % of the target volume, D_98%_) and mean dose (Dmean) were analysed. For serial OARs, such as the spinal cord and brainstem, D_2%_ and Dmean were analysed. For parallel organs, such as the rectum and bladder, Dmean was emphasized. To evaluate the overall quality of the treatment plans, the conformity index (CI) and homogeneity index (HI) were calculated as follows [21]:

$$ \mathrm{C}\mathrm{I}=\frac{{\mathrm{TV}}_{\mathrm{RI}}}{\mathrm{TV}}\times \frac{{\mathrm{TV}}_{\mathrm{RI}}}{{\mathrm{V}}_{\mathrm{RI}}} $$

Where TV_RI_ is the target volume covered by the prescription isodose, TV is the target volume, and V_RI_ is the volume of the prescription isodose. The CI ranges from 0 to 1, where 1 indicates perfect overlap (identical structures). A value near 0 indicates total absence of conformation, i.e., the target volume is not irradiated.

$$ \mathrm{H}\mathrm{I}=\frac{{\mathrm{D}}_{2\%}-{\mathrm{D}}_{98\%}}{{\mathrm{D}}_{\mathrm{prescription}}}\times 100\% $$

Where D_2%_ is the dose received by 2 % of the target volume, D_98%_ is the dose received by 98 % of the target volume, and Dprescription is the prescription dose of the target volume. The HI ranges from 0 to 1, where 0 is the ideal value. A higher HI indicates poorer homogeneity.

To evaluate dose delivery efficiency, monitor units (MUs), control points (or segments) per fraction and beam on time were compared. In addition, planning time for each plan was also considered in the evaluation of planning efficiency. In this study, we developed a protocol to define the indispensability of leaf interdigitation with different inverse planning technologies for four tumour sites. The protocol, on the basis of the ‘Traffic Light Protocol’, has three levels: level red, level yellow and level green. Superior quality plan is the primary consideration regardless of the delivery efficiency. Plan quality was assessed by an experienced radiation therapist. Delivery efficiency is another consideration if the plan quality is equivalent. We quantify delivery efficiency by assessing the scores of every index (MUs, segments, and beam on time). Regarding the leaf interdigitation plans, one point indicates high delivery efficiency. Zero point indicates equivalent delivery efficiency or low delivery efficiency. We evaluated the indispensability of leaf interdigitation by comparing the scores.

1. Level red (with): Requires leaf interdigitation. Case 1: a superior quality plan was identified regardless of the delivery efficiency. Case 2: high delivery efficiency was found when the plan quality is equivalent.
2. Level yellow (with/without): Leaf interdigitation is dispensable. The plan quality is equivalent, and the delivery efficiency are identical.
3. Level green (without): No need for leaf interdigitation. Case 1: the plan quality is inferior regardless of the delivery efficiency. Case 2: low delivery efficiency was found when the plan quality is equivalent.

### Statistical analysis

Paired Student’s t-tests or Wilcoxon rank tests were used to compare the differences between the leaf interdigitation plans and the leaf non-interdigitation plans. Paired Student's *t*-test was used when the data fit a normal distribution. If the data did not fit the normal distribution, we selected the Wilcoxon rank test instead of paired Student's *t*-test. All analyses were performed using SPSS version 16.0 (SPSS, Chicago, IL, USA). *P* < 0.05 was considered significant.

## Results

### PTV coverage

All generated plans were clinically acceptable. For the MBM patients, the comparison of leaf interdigitation plans and leaf non-interdigitation plans with respect to PTV is summarized in Table 2. Leaf interdigitation yielded equivalent results for the observed evaluation parameters in the dMLC and ssIMRT plans. For the NPC-SIB patients, leaf interdigitation provided little benefit with respect to the HI for the ssIMRT plans in spite of a large and complex target volume. In the other two cases, all evaluation variables of PTV were equivalent for leaf interdigitation plans and leaf non-interdigitation plans. The PTV comparative results of the NPC-SIB, cervical and prostate cases are summarized in the supplementary material (see Additional file 1: S1, S2 and S3).

**Table 2:** The PTV comparative results of leaf interdigitation plans and leaf non-interdigitation plans in MBM sites

	VMAT	dMLC	ssIMRT
	with-without	with-without	with-without
PTVmax(Gy)	64.9 ± 1.1/62.8 ± 0.9	65.5 ± 1.8/65.9 ± 1.5	64.7 ± 2.2/65.1 ± 2.8
PTVmin(Gy)	58.8 ± 1.5/58.5 ± 1.7*	57.9 ± 1.9/58.2 ± 1.8	57.6 ± 1.3/57.2 ± 1.5
PTVmean(Gy)	62.4 ± 0.5/62.3 ± 0.6	62.1 ± 0.9/62.6 ± 0.8	61.9 ± 1.1/62.8 ± 0.9
HI	0.09 ± 0.028/0.108 ± 0.04*	0.091 ± 0.042/0.101 ± 0.052	0.102 ± 0.091/0.102 ± 0.07
CI	0.79 ± 0.06/0.78 ± 0.05	0.75 ± 0.1/0.75 ± 0.12	0.74 ± 0.11/0.74 ± 0.15

Note: * represents statistically significant between the two sets of data

### OARs doses

Table 3 shows all of the OARs parameters for the MBM patients. The OARs comparative results for the NPC-SIB, cervical and prostate cases are summarized in the supplementary data (see Additional file 1: S4, S5 and S6). No statistically significant differences were found in any of the leaf interdigitation and leaf non-interdigitation plans.

**Table 3:** The OARs parameter values of leaf interdigitation plans and leaf non-interdigitation plans in MBM sites

	VMAT(Gy)	dMLC(Gy)	ssIMRT(Gy)
	with-without	with-without	with-without
Brainstem	37.0 ± 14.8/37.2 ± 15.1	37.8 ± 13.2/38.1 ± 16.5	37.2 ± 14.8/37.9 ± 16.2
Optic nerves-L	13.8 ± 11.6/12.9 ± 9.4	13.4 ± 9.6/13.8 ± 9.9	14.3 ± 10.5/13.5 ± 9.8
Optic nerves-R	17.4 ± 8.8/11.3 ± 9.2	17.9 ± 9.8/12.5 ± 10.3	17.5 ± 7.9/16.4 ± 9.4
Eye -L	12.7 ± 11.3/12.6 ± 10.7	12.9 ± 10.3/12.8 ± 10.2	13.3 ± 10.1/13.2 ± 10.9
Eye-R	13.2 ± 11.8/13.3 ± 13.0	14.4 ± 10.7/15.1 ± 11.0	13.3 ± 9.8/13.6 ± 9.5
Lens-L	3.6 ± 1.7/3.5 ± 1.7	3.6 ± 1.8/3.5 ± 1.5	3.7 ± 1.6/3.6 ± 1.9
Lens-R	3.5 ± 1.9/3.5 ± 1.9	3.5 ± 1.8/3.6 ± 1.6	3.6 ± 1.2/3.6 ± 1.8

Note: For brainstem, optic nerves, lens and eyes, the value represents D_2%_

### Delivery efficiency

The monitor units, segments, beam on time and planning time for each technique used in the MBM patients are shown in Table 4. We observed that leaf interdigitation generated more MUs, segments and beam on time in the dMLC plans. For the VMAT plans, leaf interdigitation showed an increase of 3.7 % compared with leaf non-interdigitation in the MUs. For the ssIMRT plans, leaf interdigitation showed decreases of 25.3 % and 19.6 % in the MUs and segments, respectively. The delivery efficiency results from the NPC-SIB, cervical and prostate cases are summarized in the supplementary data (see Additional file 1: S7, S8 and S9). All results revealed that leaf interdigitation increased the MUs and segments in the dMLC plans. However, leaf interdigitation reduced the MUs in the ssIMRT plans for NPC-SIB patients and MBM patients. We found significant differences in the segments and beam on time in the NPS-SIB patients. In addition, significant differences were also observed in the beam on time in the prostate patients. Notably, leaf interdigitation saved the planning time in the VMAT and dMLC plans. By contrast, the planning time in the leaf interdigitation protocols was increased in the ssIMRT plans.

**Table 4:** Delivery efficiency of leaf interdigitation plans and leaf non-interdigitation plans in MBM sites

	VMAT	dMLC	ssIMRT
	with-without	with-without	with-without
MUs	680 ± 54/656 ± 45*	917 ± 102/792 ± 128*	621 ± 94/831 ± 103*
Segments	169 ± 8/169 ± 8	575 ± 97/346 ± 91*	82 ± 11/102 ± 21*
BOT(s)	238 ± 19/237 ± 19	548 ± 72/460 ± 73*	545 ± 51/560 ± 54
PT(s)	17 ± 5/23 ± 4*	7 ± 2/12 ± 5*	15 ± 6/6 ± 5*

Note: *BOT* represents beam on time, *PT* represents planning time, * represents statistically significant between the two sets of data

### Conditions in which leaf interdigitation provides benefits

On the basis of these observations and the abovementioned protocol, we defined the conditions in which leaf interdigitation provides benefits. These conditions are presented in Table 5.

**Table 5:** The conditions that leaf interdigitation can generate benefits

Tumour sites	VMAT	dMLC	ssIMRT
NPC	without	without	with
MBM	with	without	with/without
Cervical	without	without	with/without
Prostate	without	without	without

Note: *with* represents needs for leaf interdigitation; *with/without* represents needs are formidable evaluation for leaf interdigitation; *without* represents no needs for leaf interdigitation

## Discussion

MLCs have significant efficacy and efficiency in radiotherapy. They are now widely applied and have become integral parts of every radiotherapy department. In 3D-CRT and IMRT technologies, MLCs play a critical role in generating conformal shaping and providing the intensity-modulated beam. The characteristics and dosimetry issues associated with MLCs are crucial for optimal treatment quality and treatment safety and efficiency.

In this report, we present an experimental study of leaf interdigitation with different inverse planning techniques for representative tumour sites. To keep the bias of the study low, one expert planner generated the leaf interdigitation and leaf non-interdigitation plans using the same TPS version, the same linear accelerator, the same planning parameters and the same dose algorithms. The aim of this research was to assess the dosimetric impact of leaf interdigitation on multiple inverse planning techniques for representative tumour sites. We expected to detect the conditions in which leaf interdigitation can provide benefits. The results indicated that leaf interdigitation could not improve plan quality in multiple inverse planning techniques, regardless of the complexity of the target. However, leaf interdigitation influenced delivery efficiency, particularly in the dMLC and ssIMRT plans. Furthermore, the planning time was shortened by leaf interdigitation in the VMAT and dMLC plans. It is interesting that this phenomenon did not exist in the ssIMRT plans. By contrast, leaf interdigitation increased the planning time for ssIMRT plans.

Our findings are consistent with that of the study by Kesteren et al. [17]. Their studied revealed that leaf interdigitation had a minimal dosimetric impact on prostate and rectal cancer treatment plans in VMAT treatments using the Pinnacle TPS. Lafond et al. [3] found no dosimetric advantages to using MLCi2 interdigitation for HNC in VMAT plans. Furthermore, their study showed that interdigitation could improve efficiency. In our study, we found that interdigitation increased the MUs for the MBM VMAT plans and all dMLC plans. However, this phenomenon was not found in other cases. Instead, interdigitation resulted in a decrease in MUs for all ssIMRT cases, except prostate cases. Moreover, consistent with the literature [22], we also observed that leaf interdigitation shortened the solution time for the VMAT plans.

This study reveals that leaf interdigitation can reduce VMAT and dMLC planning time. Inverse treatment plans must be repeatedly altered by cost functions, priorities and other planning parameters. The process is time-consuming, particularly in complex cases. In addition, with the growing popularity of VMAT and dMLC for radiation therapy, there is a greater need for improving the planning efficiency. Our results demonstrated that leaf interdigitation could increase the dosimetrist efficiency by saving planning time in the design of VMAT and dMLC treatment plans.

MLC leaf interdigitation can create island fields in complex situations, whereby one trailing leaf advances beyond the position of an adjacent leading leaf. For the CRT plans of multiple tumours, leaf interdigitation is commonly applied to obtain reasonable distributions. Leaf interdigitation is forbidden in inverse treatment plans in early types of MLCs with an Elekta linear accelerator. Then, the suppliers adopted the MLCi2 MLCs, which allowed interdigitation on inverse treatment plans. Theoretically, leaf interdigitation increases the degrees of freedom for better treatment plans. However, we did not obtain superior quality plans by leaf interdigitation in this study, except for small significant differences in a few cases. The process of inverse planning optimization entails searching for the local optimum in the solution space, although the global optimum is difficult to identify. In principle, non-interdigitation MLC treatment plan optimization solutions are a sub-set of interdigitation MLC plan optimization solutions. Because of the addition of more degrees of freedom, it is possible that leaf interdigitation obtains more paths to search for the local optimum than leaf non-interdigitation [17]. Thus, leaf interdigitation can save time in the search for the local optimum. This may explain the shortened planning time results observed in this study for the VMAT and dMLC plans. However, we found that the planning time was increased for the ssIMRT plans. Likely, there may be a balance between the search space and search path. The sliding window technique used for dMLC and VMAT may not suffer as large a discrepancy between search space and search path as the ssIMRT-sequencing algorithm when changing from non-interdigitation to interdigitation.

Utilizing the modulation of leaf movement, the number of fields, dose rate and other parameters, balance was reached between the target volume coverage and the OARs doses in inverse planning technologies. Leaf interdigitation is an intensity modulation method for inverse planning strategies. In this study, we did not acquire higher quality plans. It is possible that the local optimum in the solution space does not require leaf interdigitation because other intensity modulation methods can offset the lack of leaf interdigitation. However, leaf interdigitation may change the paths of the optimization solutions. Consequently, the MUs, segments and delivery time may effect changes. As an example, leaf interdigitation splits the segments and generates a greater number of smaller and narrower segments in dMLC plans [23]. Therefore, leaf interdigitation dMLC plans lead to more segments than leaf non-interdigitation dMLC plans. In addition, we conclude that the optimization algorithm in the Monaco TPS may influence the MUs and delivery time. This assumption should be confirmed in future studies.

Our study demonstrates that leaf interdigitation may not always generate benefits when performing inverse treatment strategies for various tumour sites. The study may provide useful guidelines for selecting reasonable planning treatment methods in current clinical practice. It should be noted that the results are related to one linac and one TPS. Different planning systems and different Linac produced by other manufacturers should be studied in future investigations to overcome the variance between treatment facilities.

## Conclusion

In this report, the effect of MLC leaf interdigitation on inverse treatment plans was experimentally studied in multiple tumour sites. We demonstrate that leaf interdigitation leads to obvious differences in the delivery efficiency of dMLC plans and ssIMRT plans. Furthermore, leaf interdigitation may influence planning efficiency without affecting target coverage and OARs sparing. On the basis of these observations, our study suggests that leaf interdigitation should be utilized when performing MBM VMAT and NPC ssIMRT plans.

## Additional file

Additional file 1: Tables S1. The PTV comparative results of the leaf interdigitation plans and leaf non-interdigitation plans in NPC sites. Tables S2. The PTV comparative results of the leaf interdigitation plans and leaf non-interdigitation plans in cervical sites. Tables S3. The PTV comparative results of the leaf interdigitation plans and leaf non-interdigitation plans in prostate sites. Tables S4. The OARs parameter values of the leaf interdigitation plans and leaf non-interdigitation plans in NPC sites. Tables S5. The OARs parameter values of the leaf interdigitation plans and leaf non-interdigitation plans in cervical sites. Tables S6. The OARs parameter values of the leaf interdigitation plans and leaf non-interdigitation plans in prostate sites. Table S7. Delivery efficiency of the leaf interdigitation plans and leaf non-interdigitation plans in NPC sites. Table S8. Delivery efficiency of the leaf interdigitation plans and leaf non-interdigitation plans in cervical sites. Table S9. Delivery efficiency of the leaf interdigitation plans and leaf non-interdigitation plans in prostate sites. (DOCX 23 kb)

## Abbreviations

3D: Three dimensional
CI: conformity index
CRT: conformal radiation therapy
CT: computed tomography
CTV: clinical target volume
dMLC: sliding window IMRT
DVH: dose volume histogram
HI: homogeneity index
IMRT: intensity modulated radiotherapy
MBM: multiple brain metastasis
MLC: multileaf collimator
MU: monitor units
NPC: nasopharyngeal carcinoma
OARs: organs at risk
PTV: planning target volume
SIB: simultaneous integrated boost
ssIMRT: step-and-shoot IMRT
TPS: treatment planning system
VMAT: volumetric modulated arc therapy

## References

[1] DeVita VT, Rosenberg SA (2012). Two hundred years of cancer research. New Engl J Med.

[2] Hong CS, Ju SG, Kim M, Kim JI, Kim JM, Suh TS (2014). Dosimetric effects of multileaf collimator leaf width on intensity-modulated radiotherapy for head and neck cancer. Med Phys.

[3] Lafond C, Chajon E, Devillers A, Louvel G, Toublanc S, Olivier M (2013). Impact of MLC leaf width on volumetric-modulated arc therapy planning for head and neck cancers. J Appl Clin Med Phys.

[4] Yoganathan SA, Mani KR, Das KJM, Agarwal A, Kumar S (2011). Dosimetric effect of multileaf collimator leaf width in intensity-modulated radiotherapy delivery techniques for small-and large-volume targets. J Med Phys.

[5] Wu QJ, Wang Z, Kirkpatrick JP, Chang Z, Meyer JJ, Lu M (2009). Impact of collimator leaf width and treatment technique on stereotactic radiosurgery and radiotherapy plans for intra-and extracranial lesions. Radiat Oncol.

[6] Fiveash JB, Murshed H, Duan J, Hyatt M, Caranto J, Bonner JA (2002). Effect of multileaf collimator leaf width on physical dose distributions in the treatment of CNS and head and neck neoplasms with intensity modulated radiation therapy. Med Phys.

[7] Chang J, Yenice KM, Jiang K, Hunt M, Narayana A (2009). Effect of MLC leaf width and PTV margin on the treatment planning of intensity-modulated stereotactic radiosurgery (IMSRS) or radiotherapy (IMSRT). Med Dosim.

[8] Chae SM, Lee GW, Son SH (2014). The effect of multileaf collimator leaf width on the radiosurgery planning for spine lesion treatment in terms of the modulated techniques and target complexity. Radiat Oncol.

[9] Serna A, Puchades V, Mata F, Ramos D, Alcaraz M (2015). Influence of multi-leaf collimator leaf width in radiosurgery via volumetric modulated arc therapy and 3D dynamic conformal arc therapy. Phys Medica.

[10] Vorwerk H, Wagner D, Hess CF (2008). Impact of different leaf velocities and dose rates on the number of monitor units and the dosevolume-histograms using intensity modulated radiotherapy with sliding-window technique. Radiat Oncol.

[11] Deng J, Pawlicki T, Chen Y, Li J, Jiang SB, Ma CM (2001). The MLC tongue-and-groove effect on IMRT dose distributions. Phys Med Biol.

[12] Cozzi L, Dinshaw KA, Shrivastava SK, Mahantshetty U, Engineer R, Deshpande DD (2008). A treatment planning study comparing volumetric arc modulation with RapidArc and fixed field IMRT for cervix uteri radiotherapy. Radiother Oncol.

[13] Vanetti E, Clivio A, Nicolini G, Fogliata A, Ghosh-Laskar S, Agarwal JP (2009). Volumetric modulated arc radiotherapy for carcinomas of the oro-pharynx, hypo-pharynx and larynx: a treatment planning comparison with fixed field IMRT. Radiother Oncol.

[14] Canyilmaz E, Uslu GDH, Colak F, Hazeral B, Haciislamoglu E, Zengin AY (2015). Comparison of dose distributions hippocampus in high grade gliomas irradiation with linac-based imrt and volumetric arc therapy: a dosimetric study. SpringerPlus.

[15] Ehrgott M, Güler Ç, Hamacher HW, Shao LZ (2008). Mathematical optimization in intensity modulated radiation therapy. 4OR.

[16] Kalinowski T (2008). Reducing the tongue–and–groove underdosage in MLC shape matrix decomposition. Algorithmic Operations Research.

[17] van Kesteren Z, Janssen TM, Damen E, van Vliet-Vroegindeweij C (2012). The dosimetric impact of leaf interdigitation and leaf width on VMAT treatment planning in Pinnacle: comparing Pareto fronts. Phys Med Biol.

[18] Kantz S, Söhn M, Troeller A, Reiner M, Weingandt H, Alber M (2015). Impact of MLC properties and IMRT technique in meningioma and head-and-neck treatments. Radiat Oncol.

[19] Tacke MB, Nill S, Häring P, Oelfke U (2008). 6 MV dosimetric characterization of the 160 MLC™, the new Siemens multileaf collimator. Med Phys.

[20] Sharfo AWM, Voet PWJ, Breedveld S, Mens JWM, Hoogeman MS, Heijmen BJ (2015). Comparison of VMAT and IMRT strategies for cervical cancer patients using automated planning. Radiother Oncol.

[21] Feuvret L, Noël G, Mazeron JJ, Bey P (2006). Conformity index: a review. Int J Radiat Oncol Biol Phys.

[22] Peng F, Jia X, Gu X, Epelman MA, Romeijn HE, Jiang SB (2012). A new column-generation-based algorithm for VMAT treatment plan optimization. Phys Med Biol.

[23] Ning ZH, Mu JM, Jin JX, Li XD, Li QL, Gu WD (2013). Single arc volumetric-modulated arc therapy is sufficient for nasopharyngeal carcinoma: a dosimetric comparison with dual arc VMAT and dynamic MLC and step-and-shoot intensity-modulated radiotherapy. Radiat Oncol.

