# Supplementary material for: Capability of leaf interdigitation with different inverse planning strategies in Monaco: an investigation of representative tumour sites
Source: Radiat Oncol. 2016 Jun 17;11:82. doi: 10.1186/s13014-016-0655-1 (PMC4912757; doi:10.1186/s13014-016-0655-1)
Supplement: Additional file 1: Tables S1. — The PTV comparative results of the leaf interdigitation plans and leaf non-interdigitation plans in NPC sites. Tables S2. The PTV comparative results of the leaf interdigitation plans and leaf non-interdigitation plans in cervical sites. Tables S3. The PTV comparative results of the leaf interdigitation plans and leaf non-interdigitation plans in prostate sites. Tables S4. The OARs parameter values of the leaf interdigitation plans and leaf non-interdigitation plans in NPC sites. Tables S5. The OARs parameter values of the leaf interdigitation plans and leaf non-interdigitation plans in cervical sites. Tables S6. The OARs parameter values of the leaf interdigitation plans and leaf non-interdigitation plans in prostate sites. Table S7. Delivery efficiency of the leaf interdigitation plans and leaf non-interdigitation plans in NPC sites. Table S8. Delivery efficiency of the leaf interdigitation plans and leaf non-interdigitation plans in cervical sites. Table S9. Delivery efficiency of the leaf interdigitation plans and leaf non-interdigitation plans in prostate sites. (DOCX 23 kb) [file 13014_2016_655_MOESM1_ESM.docx]

Tables S1. The PTV comparative results of leaf interdigitation plans and leaf non-interdigitation plans in NPC sites

|  |  | VMAT | dMLC | ssIMRT |
| --- | --- | --- | --- | --- |
|  |  | with-without | with-without | with-without |
| PTVmax(Gy) | PTV_70_ | 75.9±1.4/75.9±1.8 | 76.5±1.3/76.6±1.5 | 76.6±2.1/76.5±2.2 |
|  | PTV_60_ | 68.7±1.5/68.6±1.3 | 68.9±1.3/68.9±1.7 | 68.1±1.4/68.2±1.3 |
|  | PTV_54_ | 58.7±0.9/58.8±1.1 | 58.8±1.1/58.8±0.9 | 58.8±1.0/58.8±1.2 |
| PTVmin(Gy) | PTV_70_ | 65.3±1.4/65.4±1.5 | 65.4±2.0/65.5±1.8 | 65.4±1.8/65.3±1.3 |
|  | PTV_60_ | 52.7±1.1/52.7±1.4 | 52.6±0.8/52.7±1.6 | 52.6±2.2/52.6±1.9 |
|  | PTV_54_ | 49.6±0.8/49.7±0.8 | 49.5±1.2/49.5±2.2 | 49.5±1.8/49.4±0.9 |
| PTVmean(Gy) | PTV_70_ | 73.4±1.1/73.3±1.2 | 73.8±1.5/73.8±1.2 | 73.9±2.4/73.9±2.6 |
|  | PTV_60_ | 65.5±2.2/65.6±1.9 | 65.9±1.5/65.9±1.8 | 66.1±2.3±66.2±2.8 |
|  | PTV_54_ | 56.8±2.6/56.8±1.8 | 56.9±1.2/56.8±1.1 | 56.9±1.9/56.9±1.8 |
| HI | PTV_70_ | 0.151±0.03/0.151±0.03 | 0.152±0.02/0.152±0.02 | **0.152±0.02/0.162±0.02*** |
|  | PTV_60_ | 0.261±0.05/0.261±0.04 | 0.261±0.08/0.267±0.08 | **0.262±0.07/0.271±0.06*** |
|  | PTV_54_ | 0.171±0.07/0.172±0.08 | 0.172±0.05/0.173±0.09 | **0.172±0.06/0.183±0.08*** |
| CI | PTV_70_ | 0.71±0.11/0.71±0.09 | 0.69±0.09/0.69±0.11 | 0.69±0.08/0.69±0.08 |
|  | PTV_60_ | 0.56±0.08/0.56±0.07 | 0.56±0.09/0.56±0.08 | 0.56±0.07/0.55±0.09 |
|  | PTV_54_ | 0.78±0.06/0.79±0.07 | 0.76±0.07/0.76/0.08 | 0.75±0.11/0.75±0.12 |

Note. * represents statistically signiﬁcant between the two sets of data.

Tables S2. The PTV comparative results of leaf interdigitation plans and leaf non-interdigitation plans in cervical cancer sites

|  | VMAT | dMLC | ssIMRT |
| --- | --- | --- | --- |
|  | with-without | with-without | with-without |
| PTVmax(Gy) | 54.9±1.1/54.7±1.4 | 54.9±1.3/55.1±1.5 | 54.8±1.2/54.9±1.3 |
| PTVmin(Gy) | 49.7±1.5/49.7±1.6 | 49.9±1.3/49.9±1.8 | 49.6±1.2/49.8±1.6 |
| PTVmean(Gy) | 52.6±1.4/52.5±1.3 | 52.6±1.2/52.5±1.3 | 52.6±1.3/52.5±1.6 |
| HI | 0.101±0.021/0.102±0.02 | 0.099±0.022/0.101±0.03 | 0.102±0.019/0.101±0.021 |
| CI | 0.82±0.09/0.82±0.08 | 0.81±0.11/0.81±0.14 | 0.79±0.07/0.80±0.12 |

Tables S3. The PTV comparative results of leaf interdigitation plans and leaf non-interdigitation plans in prostate cancer sites

|  | VMAT | dMLC | ssIMRT |
| --- | --- | --- | --- |
|  | with-without | with-without | with-without |
| PTVmax(Gy) | 82.5±1.9/82.6±2.1 | 82.7±3.3/82.4±4.1 | 82.1±1.6/82.5±3.2 |
| PTVmin(Gy) | 74.5±1.6/74.4±1.5 | 74.8±2.1/74.2±1.8 | 74.6±2.1/73.8±1.9 |
| PTVmean(Gy) | 80.6±2.3/81.2±2.5 | 81.1±3.3/81.2±2.4 | 80.0±2.3/81.2±2.5 |
| HI | 0.104±0.021/0.103±0.03 | 0.101±0.022/0.101±0.04 | 0.108±0.031/0.108±0.033 |
| CI | 0.84±0.06/0.84±0.05 | 0.82±0.05/0.82±0.08 | 0.83±0.05/0.82±0.04 |

Tables S4. The OARs parameter values of leaf interdigitation plans and leaf non-interdigitation plans in NPC sites

|  | VMAT(Gy) | dMLC(Gy) | ssIMRT(Gy) |
| --- | --- | --- | --- |
|  | with-without | with-without | with-without |
| Brainstem | 48.2±4.6/48.4±3.9 | 49.6±5.3/49.9±4.8 | 50.2±4.7/50.1±4.4 |
| Spinal cord | 37.5±3.2/37.8±2.4 | 38.1±4.6/37.9±4.9 | 38.8±3.4/38.6±3.7 |
| Parotid-L | 26.4±2.5/26.2±2.4 | 27.2±3.1/27.4±3.4 | 27.3±2.8/27.3±2.5 |
| Parotid-R | 26.3±3.1/26.2±2.8 | 27.2±2.8/27.3±2.4 | 27.5±3.6/27.4±3.5 |
| Eye-L | 19.4±7.2/18.9±8.8 | 21.2±9.4/21.4±8.6 | 22.1±8.7/22.5±6.9 |
| Eye-R | 18.9±7.8/19.1±8.2 | 20.2±8.7/20.7±9.4 | 21.2±8.6/21.2±9.9 |
| Lens-L | 4.5±2.5/4.5±2.6 | 5.1±1.8/5.1±1.5 | 4.9±2.0/4.8±2.2 |
| Lens-R | 4.4±2.4/4.5±2.5 | 4.8±1.6/4.9±2.8 | 5.1±1.4/4.9±2.4 |
| Optic nerves-L | 19.8±10.2/19.9±9.6 | 20.8±10.2/20.3±8.9 | 21.2±11.2/22.2±12.4 |
| Optic nerves-R | 20.5±8.8/21.1±9.4 | 22.5±11.2/22.6±11.4 | 22.9±13.1/22.8/13.5 |

Note: For brainstem, spinal cord, eyes, lens and optic nerves, the value represents D_2%_. For parotids, the value represents Dmean.

Tables S5. The OARs parameter values of leaf interdigitation plans and leaf non-interdigitation plans in cervical cancer sites

|  | VMAT(Gy) | dMLC(Gy) | ssIMRT(Gy) |
| --- | --- | --- | --- |
|  | with-without | with-without | with-without |
| Bladder | 32.9±1.7/32.6±1.6 | 34.4±1.4/34.5±1.6 | 34.5±1.5/34.5±1.7 |
| Rectum | 32.3±1.6/32.4±1.4 | 35.5±1.3/35.4±1.6 | 35.4±1.9/35.5±1.8 |
| Femur head-L | 20.2±2.2/20.2±1.9 | 22.3±2.3/22.2±1.8 | 22.7±2.5/22.6±2.2 |
| Femur head-R | 19.6±1.8/19.8±2.1 | 21.5±2.3/21.7±2.5 | 22.1±2.6/22.2±2.3 |
| Small bowel | 24.2±1.4/24.1±1.8 | 24.5±2.4/24.8±2.2 | 24.6±2.8/24.5±2.4 |

Note: For bladder, rectum, femur heads and small bowel, the value represents Dmean.

Tables S6. The OARs parameter values of leaf interdigitation plans and leaf non-interdigitation plans in prostate cancer sites

|  | VMAT(Gy) | dMLC(Gy) | ssIMRT(Gy) |
| --- | --- | --- | --- |
|  | with-without | with-without | with-without |
| Bladder | 17.5±8.3/17.2±7.5 | 17.7±5.6/17.4±6.2 | 17.4±6.6/17.8±8.5 |
| Rectum | 25.2±10.2/26.1±8.8 | 26.3±8.2/25.9±7.4 | 26.1±8.4/26.2±6.9 |
| Femur head-L | 15.9±9.2/15.8±8.1 | 16.4±8.8/16.7±6.9 | 16.2±9.4/15.9±7.7 |
| Femur head-R | 16.3±7.8/16.1±9.4 | 16.5±8.4/17.2±9.7 | 16.8±6.8/16.1±8.7 |

Note: For bladder, rectum and femur heads, the value represents Dmean.

Table S7. Delivery efficiency of leaf interdigitation plans and leaf non-interdigitation plans in NPC sites

|  | VMAT | dMLC | ssIMRT |
| --- | --- | --- | --- |
|  | with-without | with-without | with-without |
| MUs | 887±136/890±135 | **1148±85/1065±110*** | **738±95/974±173*** |
| Segments | 238±10/238±9 | **839±66/626±340*** | **101±14/137±26*** |
| BOT(s) | 311±21/320±20 | 675±42/677±60 | **761±62/825±92*** |
| PT(s) | **42±5/58±9*** | **23±9/30±7*** | **27±18/22±8*** |

Note. BOT represents beam on time; PT represents planning time; * represents statistically signiﬁcant between the two sets of data.

Table S8. Delivery efficiency of leaf interdigitation plans and leaf non-interdigitation plans in cervical cancer sites

|  | VMAT | dMLC | ssIMRT |
| --- | --- | --- | --- |
|  | with-without | with-without | with-without |
| MUs | 1096±226/1061±201 | **1246±202/1172±158*** | **924±208/1069±326*** |
| Segments | 200±19/201±19 | **775±127/727±173*** | 131±31/140±43 |
| BOT(s) | 301±28/305±31 | 727±58/720±62 | 799±116/788±129 |
| PT(s) | **42±12/51±17*** | **13±4/16±5*** | **23±12/19±14*** |

Note. BOT represents beam on time; PT represents planning time; * represents statistically signiﬁcant between the two sets of data.

Table S9. Delivery efficiency of leaf interdigitation plans and leaf non-interdigitation plans in prostate cancer sites

|  | VMAT | dMLC | ssIMRT |
| --- | --- | --- | --- |
|  | with-without | with-without | with-without |
| MUs | 827±127/840±129 | **871±126/846±130*** | 673±113/682±122 |
| Segments | 188±6/183±10 | **607±118/538±128*** | 71±4/71±7 |
| BOT(s) | 211±12/218±15 | 394±31/390±20 | **440±32/408±29*** |
| PT(s) | **26±8/31±7*** | **13±4/17±3*** | **20±6/9±3*** |

Note. BOT represents beam on time; PT represents planning time; * represents statistically signiﬁcant between the two sets of data.
